# Primary cutaneous apocrine carcinoma of the scalp: Two case reports and literature review

**DOI:** 10.1097/MD.0000000000028808

**Published:** 2022-02-11

**Authors:** Jun Ho Choi, Hyun Myung Oh, Kwang Seog Kim, Yoo Duk Choi, Sung Pil Joo, Won Joo Hwang, Jae Ha Hwang, Sam Yong Lee

**Affiliations:** a Department of Plastic and Reconstructive Surgery, Chonnam National University Medical School, Chonnam National University Hospital, Gwangju, Republic of Korea; b Department of Pathology, Chonnam National University Medical School, Chonnam National University Hospital, Gwangju, Republic of Korea; c Department of Neurosurgery, Chonnam National University Medical School, Chonnam National University Hospital, Gwangju, Republic of Korea.

**Keywords:** apocrine, carcinoma, scalp, sweat glands

## Abstract

**Rationale:**

Apocrine carcinoma is a rare malignant sweat gland tumor that has been reported in approximately 200 cases. This tumor usually occurs in the axilla, but in rare cases, it can also develop in the scalp. In the present work, we report 2 cases of cutaneous apocrine carcinoma of the scalp.

**Patient concerns:**

Two men visited our outpatient clinic with recurrence of tumor after undergoing surgery for scalp tumor at another hospital.

**Diagnoses:**

Brain magnetic resonance imaging of a 56-year old man showed the presence of a 5.0 × 4.5 × 4.4 cm scalp mass in the right parietal region, invading the skull and dura mater and a 2.2 × 2.0 × 0.7 cm bony mass without any skin lesions right next to the scalp mass. Neck magnetic resonance imaging of a 76-year-old man revealed the presence of a well-defined oval mass in the subcutaneous layer of the left occipital scalp and 2 enlarged lymph nodes in the left neck. Definite diagnoses were made postoperatively. The patients were diagnosed with cutaneous apocrine carcinoma. The diagnosis was confirmed through histopathological and immunohistochemical staining tests.

**Interventions:**

The tumors were removed with a wide safety margin and reconstructive surgery was performed.

**Outcomes:**

Additional radiotherapy or chemotherapy was performed. Follow-up more than 6 months revealed no recurrence or metastasis.

**Lessons:**

If accurate diagnosis and treatment had taken place at the initial stages of the primary cutaneous apocrine carcinoma, it would have been possible to prevent recurrence and intracranial invasion. As recurrent primary cutaneous apocrine carcinoma can become aggressive and difficult to treat, even a small mass on the scalp must be evaluated carefully and treated properly.

## 1 Introduction

Apocrine carcinoma is a rare malignant sweat gland tumor that has only been reported in approximately 200 cases.^[1]^ Apocrine carcinoma primarily develops between the ages of 60 and 70 years among Caucasians, and its cause remains unknown.^[2]^ It mainly occurs in the axilla, which has a high concentration of apocrine glands. However, in rare cases, it has been reported to develop on the scalp, anogenital region, ear canal, chest, wrist, finger, and eyelid.^[3]^ Apocrine carcinoma is an indolent, asymptomatic, cutaneous, or subcutaneous mass and its color varies from red to purple.^[4]^ In the present work, we report 2 cases of recurrent apocrine carcinoma of the scalp. As of 2021, 34 cases of primary cutaneous apocrine carcinoma (PCAC) of the scalp have been described in the articles available through PubMed and Google Scholar. Through a literature review and a presentation of 2 cases, herein, we present an analysis of apocrine carcinoma that selectively develops on the scalp in terms of its size, gross appearance and characteristics, symptoms, immunohistochemical markers, treatment, recurrence trends, metastases, and outcomes.

## 2 Case presentation

This study was approved by our Institutional Review Board and conducted in accordance with the principles of the Declaration of Helsinki. Informed written consent was obtained from both patients for publication of this report and accompanying images.

### 2.1 Case 1

**Figure 1:**
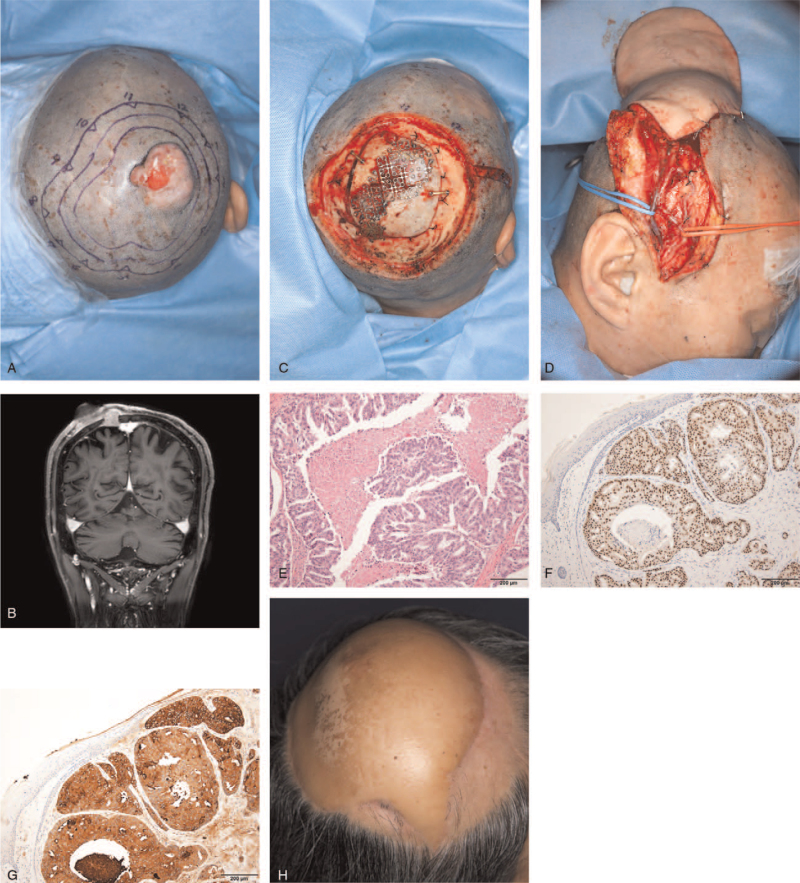
A 56-year-old man was diagnosed with apocrine carcinoma on the scalp and the mass was accompanied by a painless, reddish ulcer. (A) Preoperative photograph. (B) T1 magnetic resonance imaging of the brain showing a 5.0 × 4.5 × 4.4 cm scalp mass in the right parietal region, invading the skull and dura mater and a 2.2 × 2.0 × 0.7 cm bony mass without any skin lesions right next to the scalp mass. (C) Intraoperative photograph. The scalp mass and the skin above the bony mass were removed with a safety margin of 3 cm. After the areas of parietal bone and dura mater with tumor cell involvement were removed, duroplasty was done with artificial dura and cranioplasty was performed with a titanium mesh plate. (D) Intraoperative photograph showing scalp reconstruction using latissimus dorsi myocutaneous free flap. (E) Specimen showing the presence of the tumor with papillary architecture with central necrosis and tumor cells that have abundant eosinophilic cytoplasm with vesicular nuclei and prominent nucleoli (H&E, ×100). Immunohistochemical staining showing that the tumor cells were positive for (F) gross cystic disease fluid protein-15 (×100) and (G) androgen receptor (×100). (H) Six-month postoperative photograph.

A 56-year-old man presented to our outpatient clinic with a 4.0 × 3.5 × 3.0 cm mass on the scalp. The scalp mass, which had first been discovered 6 years back and treated with laser at another clinic, recurred 5 years later. It was surgically removed at another clinic, and the histopathological examination reported syringocystadenocarcinoma papilliferum and squamous cell carcinoma. No additional treatment was performed, and the scalp mass grew again within a year. The round mass was rigidly raised and accompanied by a painless, reddish ulcerative lesion (Fig. 1A). Brain magnetic resonance imaging showed the presence of a 5.0 × 4.5 × 4.4 cm scalp mass in the right parietal region, invading the skull and dura mater and a 2.2 × 2.0 × 0.7 cm bony mass without any skin lesions right next to the scalp mass (Fig. 1B). Positron emission tomography-computed tomography showed no metastasis to other organs. Under general anesthesia, the bony mass without any skin lesions was identified in the skull using a navigator. The scalp mass and the skin above the bony mass were removed with a safety margin of 3 cm (Fig. 1C). Subsequently, the areas of parietal bone and dura mater with tumor cell involvement were removed by the surgeons at the neurosurgery department. Duroplasty was done with artificial dura and cranioplasty was performed with a titanium mesh plate (Fig. 1C). The soft-tissue defect in the scalp was reconstructed using a latissimus dorsi myocutaneous free flap (Fig. 1D). The histopathological examination of the specimen revealed a papillary architecture with central necrosis and showed the presence of tumor cells with prominent nucleoli, vesicular nuclei, and abundant eosinophilic cytoplasm (Fig. 1E). Immunohistochemical staining of the specimen showed positive findings for gross cystic disease fluid protein-15 (GCDFP-15), cytokerain 7 (CK7), and androgen receptor (AR), and negative findings for estrogen receptor (ER) and progesterone receptor (PR) (Fig. 1F and G). Based on the combination of histopathological findings and immunohistochemical studies, the final diagnosis was apocrine carcinoma. Perineural invasion was observed in the microscopic findings. Subsequently, radiation therapy was conducted. The follow-up at 1.5 years revealed no recurrence or metastasis (Fig. 1H).

### 2.2 Case 2

**Figure 2:**
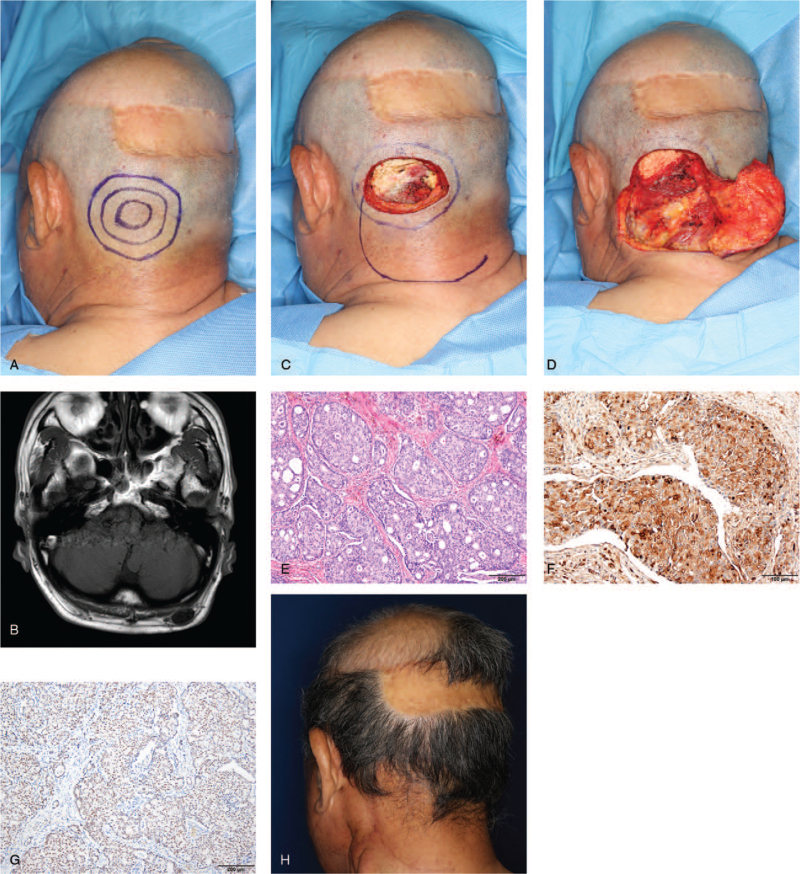
A 73-year-old man was diagnosed with apocrine carcinoma on the occipital area. (A) Preoperative photograph. (B) T1 magnetic resonance imaging of the neck showing a 1.9 × 1.6 × 0.9 cm, oval, well-defined, and non-enhancing nodular mass in the subcutaneous layer of the left occipital scalp. (C) Intraoperative photograph. Excision was done with a 2-cm safety margin, including the periosteum. (D) Intraoperative photograph showing an elevated rotation flap. (E) Specimen showing an infiltrative tumor border in the peripheral portion of the tumor (H&E, ×100). Immunohistochemical staining showing that the tumor cells were positive for (F) gross cystic disease fluid protein-15 (×100) and (G) androgen receptor (×100). (H) Six-month postoperative photograph.

A 73-year-old man visited our outpatient clinic with the complaint of a newly developed occipital mass (Fig. 2A). The patient had previously undergone excision of a mass on the left parietal scalp 3 times. The mass on the left parietal area, which was initially observed 25 years back, was first excised at another clinic 10 years back and the histopathological examination reported metastatic adenocarcinoma. The primary tumor site was never identified. Two years later, the patient visited the neurosurgery outpatient clinic at our hospital due to the recurrence of mass on the previous surgical site. The imaging examination confirmed that there was no metastasis to other organs. The mass was treated with marginal excision, and the biopsy confirmed the recurrence of metastatic adenocarcinoma. Two years later, the patient visited the plastic surgery outpatient clinic with the complaint of another recurrence of the parietal scalp mass that had increased in size to 3.0 × 2.5 cm. The mass was removed from the periosteal layer with a safety margin of 2 cm and the defect was reconstructed with a rotation flap and skin graft. The histopathological examination confirmed that it was a purely differentiated carcinoma of skin adnexal origin. After surgery, the patient received radiotherapy on the left parietal scalp. Three years later, a new mass appeared in the left occipital area, just 10 cm below the first mass without any skin lesions. Neck magnetic resonance imaging revealed the presence of a 1.9 × 1.6 × 0.9 cm well-defined oval mass without contrast enhancement in the subcutaneous layer of the left occipital scalp and 2 enlarged lymph nodes were suspected to be metastatic lymphadenopathy in the left neck (Fig. 2B). Positron emission tomography-computed tomography showed no metastasis to distant organs other than the neck. The occipital mass was removed from the periosteal layer with a safety margin of 2 cm and the defect was reconstructed with a rotation flap (Fig. 2C and D). Modified radical neck dissection was done on the left side of the neck. Histopathological examination confirmed the presence of an apocrine carcinoma with metastatic involvement of 3 cervical lymph nodes (Fig. 2E). On immunohistochemical staining, the tumor cells were confirmed positive for GCDFP-15, CK7, and AR (Fig. 2F and G). Perineural invasion was observed in the microscopic findings. The patient received adjuvant radiotherapy on the occipital area and neck, followed by chemotherapy, which included 6 cycles of cisplatin administration. The follow-up after 6 months revealed no recurrence or metastasis (Fig. 2H).

## 3 Discussion

The incidence of scalp tumors among skin tumors is gradually increasing.^[5]^ The malignancy rate of scalp tumors lies between 1% and 2%. Most malignant tumors of the scalp are basal cell carcinoma and squamous cell carcinoma.^[6]^ PCAC is a very rare malignant tumor that has been reported only in about 200 cases until date, and only 34 cases with the development on the scalp have been published in PubMed and Google Scholar as of 2021.

Primary cutaneous apocrine carcinoma is often mistaken for other types of tumors.^[7,8]^ In our case 1, the primary mass recurred after laser treatment, and the recurrent mass was removed at another clinic. At the time of removal, the specimen was misdiagnosed as syringocystadenocarcinoma papilliferum; hence, the patient did not receive any further treatment. However, a second opinion was requested regarding the pathology of the specimen, and the case was confirmed as apocrine carcinoma. In our case 2, the masses on the parietal scalp were assumed to be metastatic adenocarcinoma; however, the primary site of the cancer was not identified. The mass on the occiput was diagnosed as primary cutaneous apocrine carcinoma and was considered as an independent tumor because it occurred about 10 cm distant from the previous parietal tumor. To date, there have been 4 cases of primary apocrine carcinoma that were initially misdiagnosed as metastatic adenocarcinoma where the primary site could not be identified.^[9–12]^ Considering the difficulty in distinguishing between metastatic adenocarcinoma and PCAC, it is likely that the parietal masses are apocrine carcinomas.

PCAC often exhibits a similar form of metastasis to that of breast adenocarcinoma. Therefore, these 2 conditions need to be distinguished through a careful examination of the clinical history and biopsy.^[10]^ PCAC is characterized by the presence of cells with abundant eosinophilic cytoplasm, eccrine basally located nuclei, and decapitation secretions that are generally observed in luminal cells.^[13]^ PCAC stains positive for GCDFP-15, which stains better in apocrine glands than in eccrine glands, and for AR. On the contrary, metastatic breast adenocarcinoma is often positive for ER and PR.^[13]^ There have been reports that mammaglobin yields better staining in PCAC than in breast adenocarcinoma, and that these 2 tumors can be distinguished based on molecular studies.^[14,15]^ In the reported cases, the probability of metastasis from a primary breast lesion was low as all the patients were men and the tumors were negative for ER and PR.

The standard treatment for apocrine carcinoma is surgical excision with or without lymph node dissection. Although the surgical margin has not been standardized due to insufficient data, 1 to 2 cm can ensure sufficient eradication of the tumor cells.^[16]^ Chemotherapy and radiotherapy have been used to treat the tumor, even though the usefulness of these treatments is unproven.^[16]^ In our case 1, lymph node dissection and chemotherapy were not performed due to the absence of metastasis to other organs or lymph nodes. However, the scalp tissue was removed widely, with a 3-cm safety margin, due to the history of recurrence twice. The surgeons at the neurosurgery department sufficiently removed the areas of the skull and dura mater that showed tumor cell involvement. However, the perineural invasion was confirmed based on the microscopic findings, and radiotherapy was performed. In our case 2, cervical lymph node metastasis was confirmed by modified radical neck dissection and perineural invasion was confirmed by microscopic findings. Consequently, chemotherapy and radiotherapy were performed.

The scalp is a structure that protects the cranial bones and brain. Since the scalp tissue contains hair, which is important from a cosmetic standpoint, it is challenging to successfully reconstruct a scalp defect after removing a tumor as many different factors must be considered.^[17]^ Many methods of scalp reconstruction exist, but skin grafts are not recommended for lesions requiring radiation therapy after surgery.^[18]^ In our case 1, the scalp defect was large and the possibility of radiation therapy was high due to the invasion of the dura mater. Therefore, the defect was reconstructed using a myocutaneous free flap, which is sufficiently stable for complications induced by radiation and can cover a wide range of defects.

If accurate diagnosis and treatment had taken place at the initial stages of the PCAC, it would have been possible to prevent recurrence and intracranial invasion. Recurrent PCAC can become aggressive and difficult to treat; therefore, even the smallest mass on the scalp must be evaluated carefully and treated properly.

## 4 Literature review

We conducted a literature review of PCAC cases on the scalp in the articles that were available through PubMed and Google Scholar up to 2021. The review mainly focused on the tumor's size, gross appearance and characteristics, symptoms, immunohistochemical markers, treatment, recurrence trends, metastases, and prognostic outcomes.

In total, 36 cases had detailed reports that were gathered and condensed in Table 1.^[1,3,4,7–12,14,19–33]^ Of the 36 cases obtained, 16 were in female patients and 20 were in male patients. The average age at the time of diagnosis was 60.8 years and it ranged from 20 to 85 years. The tumors were measured based on the long axis, and ranged from 0.3 to 11 cm; the average size of the tumors was 2.97 cm.

**Table 1:** Clinical data of 36 case reports of primary cutaneous apocrine carcinoma of the scalp.

Case report	Age, y	Sex	Tumor size, cm	Metastasis at diagnosis	Surgical treatment	Chemotherapy or radiotherapy	Recurrence or metastasis	Further treatment	Outcome (follow-up)
Domingo and Helwig (1979) ^[19]^	77	M	2 × 1.2	–	Excision (excised tissue size: 3 × 1.5 cm)	–	After 6 months, right cervical lymph node. After 1.5 years, local recurrence of the lesion	Excision. NR	AWD (1.5 years)
Domingo and Helwig (1979) ^[19]^	63	F	1.5	–	Excision (excised tissue size: 4.5 × 1.8 cm)	–	–	–	NED (6 years)
Domingo and Helwig (1979) ^[19]^	69	F	0.7	–	Excision	–	–	–	LTF
Domingo and Helwig (1979) ^[19]^	65	M	7	–	Excision	–	After 6 months, left postauricular, cervical (3 cm), and supraclavicular lymph node. After 9 months, T-8 vertebral body, sacroiliac area and left pelvic bone	Cervical lymph node excision and radiotherapy. Radiotherapy	DWD (2 years)
Paties et al (1993) ^[3]^	85	M	3.5	–	Excision	–	After 2 years, cervical lymph node	NR	DOC (2.5 years)
Jacyk et al (1998) ^[20]^	54	F	4 × 1	No	Excision	–	–	–	NED (1 year)
Hwang et al (2000) ^[8]^	60	M	4 × 3	–	Excision	–	After 4 years, right retroauricular area (2.5 × 2.5, 3 × 3 cm) and left lung	–	DOC (6 years)
Morabito et al (2000) ^[21]^	46	F	–	–	Excision	–	After 4 months, right temporal scalp, and cervical lymph node. After 9 months, new local relapse. After 4 months, right temporal and parietal scalp and right cervical lymph node	Radical excision, cervical lymphadenectomy, chemotherapy (cisplatin, 5-fluorouracil), radiotherapy. Di Bella multitherapy. Systemic chemotherapy (methotrexate, bleomycin), further chemotherapy (bleomycin)	DOC (28 months)
Dalle et al (2003) ^[22]^	66	M	0.8, 0.3	–	Excision	–	–	–	R
Shimato et al (2006) ^[23]^	48	M	5	Right cervical lymph node	Wide excision (2 cm free margin), wide dissection of cervical lymph node	–	After 4 years, lung. After 2 years, right frontal lobe. Left occipital lobe. After 8 months, left occipital lobe mass aggravation	Chemotherapy (doxorubicin, etoposide, docetaxel). Excision. Gamma Knife surgery. Excision	DWD (8 years)
Robson et al (2008) ^[24]^	73	F	0.5	–	Excision	–	–	–	LTF
Robson et al (2008) ^[24]^	63	F	2.4	–	Excision	–	–	–	LTF
Robson et al (2008) ^[24]^	70	F	1.9	–	Excision	–	–	–	NED (2.5 years)
Robson et al (2008) ^[24]^	43	F	7.5	–	Excision	–	–	–	DWD (6 years)
Robson et al (2008) ^[24]^	31	M	1.4	–	Excision	–	–	–	LTF
Tlemcani et al (2010) ^[25]^	20	M	–	–	Excision	–	After 16 months, frontal scalp, left postauricular, left parotid lymph node, lung, right clavicular head and left ankle. After 39 months, scalp and brain	Zoledronic acid, pailliative radiotherapy, chemotherapy (paclitaxel, carboplatin). NR	DWD (55 months)
Kim et al (2012) ^[10]^	60	F	2.0 × 1.5	–		Chemotherapy	After 7 years, scalp (3 × 2 cm)	Wide excision (2 cm free margin), rotation flap, skin graft	NED (8 years)
Paudel et al (2012) ^[26]^	45	M	2 × 2	–	Excision, skin graft	–	–	–	LTF
Vucinić et al (2012) ^[4]^	65	F	4	Left cervical lymph node	Wide excision (2 cm free margin), skin graft, extended radical neck dissection	Chemotherapy (cisplatin, 5-fluorouracil). Radiotherapy (scalp, neck)	After 10 months, left retroauricular, cervical lymph node. After 2 months, recurrent tumor and anterior cervical lymph node. After 4 months, left parieto-occipital scalp, retromandibular, parotid lymph node, porta hepatis, hepatoduodenal ligament, lung, left iliac bone, right shoulder, and L5 vertebra	Excision, rotation flap, selective neck dissection chemotherapy (paclitaxel, carboplatin). Tumor re-excision and selective neck dissection, chemotherapy (paclitaxel, carboplatin). Bisphosphonate and supportive therapy (ibandronic acid)	DWD (3 years)
Hidaka et al (2012) ^[27]^	62	M	4.5 × 4	Cervical lymph node	Excision (3 cm free margin)	Chemotherapy (cisplatin, 5-fluorouracil). Radiotherapy (scalp, neck)	After 5 months, liver. After 7 months, liver	Chemotherapy (trastuzumab). Chemotherapy (lapatinib, capecitabine)	NED (22 months)
Arden et al (2014) ^[28]^	67	F	2.4	No	Excision, re-excision (2 cm free margin), rotation flap	–	–	–	NED (3 months)
Brown et al (2016) ^[9]^	42	F	3 × 2	No	Excision	–	–	–	NED (39 months)
Fukasawa-Momose et al (2016) ^[11]^	36	F	1 × 1	–	Excision with a wide margin, FTSG	–	–	–	NED (30 months)
Broshtilova and Gantcheva (2017) ^[29]^	72	M	3	No	Recommended (excision, en-bloc lymph node dissection)	–	–	–	NR
Al-Hakami et al (2019) ^[1]^	56	M	3 × 3	No	Complete excision, skin graft	–	After 1.5 years, right cervical lymph node (2 × 2 cm)	Modified radical neck dissection, adjuvant radiotherapy	NED (2 years)
Elefteriou-Kokolis et al (2018) ^[12]^	66	M	4	–	–	Radiotherapy (scalp)	–	–	LTF
Edgar et al (2018) ^[30]^	76	F	2.5	–	Excision, rhomboid flap, FTSG, STSG	–	–	–	NED (10 months)
Portelli et al (2020) ^[31]^	59	M	0.3	–	Excisional biopsy	–	–	–	LTF
Portelli et al (2020) ^[31]^	71	M	0.4	No	Excisional biopsy	–	–	–	NED (74 months)
Portelli et al (2020) ^[31]^	68	F	1	–	Incisional biopsy	–	–	–	LTF
Lee et al (2020) ^[32]^	66	M	1.5	–	Recommended (wide excision, sentinel lymph node biopsy)	–	–	–	NR
Popović et al (2021) ^[33]^	80	M	10 × 7 3	No	Wide excision (2 cm free margin), transposition flap, STSG	–	–	–	NED (1 year)
Balasubramanian et al (2021) ^[7]^	66	M	11 × 7.5 × 4	No	Excision, rotation advancement flap, STSG	–	–	–	NED (2 months)
DeCoste et al (2021) ^[12]^	72	F	1.2	–	Excision, re-excision (due to lymphovascular invasion)	–	–	–	NR
Choi et al (2021)	55	M	–	–	Excision	–	After 1 year, right parietal scalp (5.0 × 4.5 × 4.4 cm), skull and duramater (2.2 × 2.0 × 0.7 cm)	1) Wide excision (3 cm safety margin), duroplasty, cranioplasty, free flap, radiotherapy	NED (2 years)
Choi et al (2021)	73	M	1.9 × 1.6 × 0.9	Left cervical lymph node	Wide excision (2 cm), rotation flap, marginal lymph node dissection	Chemotherapy (cisplatin), radiotherapy (scalp, neck)	–	–	NED (1 year)

AWD = alive with disease, DOC = died with other causes, DWD = died with disease, F = female, FTSG = full thickness skin graft, LTF = lost to follow-up, M = male, NED = no evidence of disease, NR = not reported, R = refuse follow-up, STSG = split thickness skin graft.

Of the 36 reported cases, 20 elaborated on the characteristics and symptoms induced by the tumor. Of the 36 cases reported, 20 developed characteristics and symptoms induced by the tumor. The following features were described in the aforementioned articles: firmness (5); painlessness (5); red color (5); ulceration (4); plaque (4); hairlessness (4); bleeding (3); erythema (3); multiple nodules (2); granular appearance (2); polypoid appearance (1); keloid-like appearance (1); induration (1); raised appearance (1); papule (1); exophytic appearance (1); exudation (1); pus discharge (1); malodor (1); crust (1); pink color (1); pearliness (1); palpability (1); roughness (1); and mild itching (1).

Information was provided on immunohistochemical staining in 23 cases; GCDFP-15, carcinoembryonic antigen, CK7, ER, PR, epithelial membrane antigen, CK20, AR, and p63 were the most commonly expressed markers. GCDFP-15, CK7, epithelial membrane antigen, and AR staining showed positive results in most of the cases with tumors. On the contrary, CK20 and thyroid transcription factor-1 staining were negative even when the tumor was diagnosed. ER and PR staining exhibited both positive and negative results (Table 2).

**Table 2:** Immunohistochemistry data of 23 case reports of primary cutaneous apocrine carcinoma of the scalp (the table only lists markers that were reported in at least 3 cases).

Immunohistochemistry test	+	−	+/−	Sum
GCDFP-15	13	1	1	15
CEA	8	5	0	13
CK7	13	0	0	13
ER	7	6	0	13
PR	6	5	0	11
EMA	9	1	0	10
CK20	1	9	0	10
AR	8	0	1	9
p63 (tumor protein 63)	2	4	1	7
TTF-1	0	7	0	7
S100 (S100 protein)	3	3	0	6
CK5/6	2	2		4
HER2	1	3	0	4
GATA3 (GATA-binding protein 3)	4	0	0	4
AE1/3 (pan cytokeratin antibody 1/3)	3	0	0	3
Mammaglobin	3	0	0	3
SOX-10 (SRY-related HMG-box 10)	0	2	1	3
Chromogranin	1	2	0	3

AR = androgen receptor, CEA = carcinoembryonic antigen, CK = cytokeratin, EMA = epithelial membrane antigen, ER = estrogen receptor, GCDFP-15 = gross cystic disease fluid protein-15, HER2 = human epidermal growth factor receptor 2, HMG-box = high mobility group-box, PR = progesterone receptor, SRY = sex-determining region Y, TTF-1 = thyroid transcription factor-1.

Thirty-four cases were initially treated with surgical excision with or without chemoradiation therapy, which included palliative chemotherapy in 1 case. A patient who received only radiation therapy was reported in 1 case. The surgical safety margin was 1 to 3 cm. Of the entire cohort, 4 cases showed lymph node metastasis at the time of diagnosis, and recurrence or metastasis occurred in 12 cases. The occurrence of metastasis was observed mainly in the distant lymph nodes, lungs, and bones. Brain and liver metastases were reported in 2 cases each. Our case 1 is significant in the context of the patients heretofore reported, since he presented with apocrine carcinoma that had recurred twice and grown up to 5.0 cm, thereby exhibiting aggressive destruction of the skull with intracranial invasion.

Other tumors identified through microscopic findings, along with apocrine carcinoma included nevus sebaceus, syringocystadenoma papilliferum, basal cell carcinoma, cylindroma, trichoblastoma, syringoma, eccrine hydrocystoma, and squmous cell carcinoma (Table 3).^[3,8,10,19–22,26,29,30]^ In 4 cases, however, the tumor was initially diagnosed as metastatic adenocarcinoma but the primary location of the tumor was never identified (Table 3).^[9–12]^ In those cases, the tumor was later diagnosed as PCAC. Although PCAC in the scalp is a very rare tumor, considering the incidence of misdiagnosis, the number of cases may be higher than it appears.

**Table 3:** Data on other accompanying tumors from 18 case reports of primary cutaneous apocrine carcinoma of the scalp.

Variable	Cases
Other accompanying tumor	
Nevus sebaceus^[19,20,22,26,30]^	8
Syringocystadenoma papilliferum^[8,19,21]^	3
Basal cell carcinoma^[10,19]^	3
Cylindroma^[3,29]^	2
Trichoblastoma^[22,30]^	2
Syringoma^[8]^	1
Eccrine hydrocystoma^[8]^	1
Squmous cell carcinoma	1
Confused with metastatic adenocarcinoma with unknown original tumor sites^[9–12]^	4

The number of known cases where PCAC developed on the scalp is so small that its prognostic outcomes cannot be projected in a generalizable sense. In our cases, the patients demonstrated no recurrence of tumors by 24 months after surgery, and this survival trend aligns with previous reports. The difficulty in diagnosing PCAC makes it particularly challenging to collect proper data to reinforce the previous findings. However, even with just a few cases reported, it is possible to obtain a general overview of the phenomenon of PCAC on the scalp. Thus, further research and more cases are needed to develop a solid, evidence-based guideline for treating PCAC on the scalp.

## Author contributions

**Conceptualization:** Kwang Seog Kim.

**Data curation:** Jun Ho Choi, Hyun Myung Oh, Won Joo Hwang.

**Formal analysis:** Yoo Duk Choi, Sung Pil Joo, Jae Ha Hwang, Sam Yong Lee.

**Investigation:** Jun Ho Choi, Yoo Duk Choi, Sung Pil Joo, Won Joo Hwang.

**Methodology:** Yoo Duk Choi, Sung Pil Joo, Jae Ha Hwang, Sam Yong Lee.

**Project administration:** Kwang Seog Kim.

**Writing – original draft:** Jun Ho Choi, Hyun Myung Oh, Kwang Seog Kim.

**Writing – review & editing:** Kwang Seog Kim.
